# ANIMATE: Unsupervised Attributed Graph Anomaly Detection with Masked Graph Transformers

**DOI:** 10.3390/s26103176

**Published:** 2026-05-17

**Authors:** Jingtao Hu, Yi Zhang, Chengzhang Zhu, Changsheng Hou

**Affiliations:** 1 Academy of Military Sciences, Beijing 100091, China; hujingtao17@nudt.edu.cn (J.H.); kevin.zhu.china@hotmail.com (C.Z.); 2 School of Computer, National University of Defense Technology, Changsha 410073, China; zhangy@nudt.edu.cn

**Keywords:** graph anomaly detection, unsupervised graph representation learning, masked autoencoder, transformers

## Abstract

Attributed graphs have recently emerged as a powerful tool for representing diverse data in numerous real-world sensors. Among various applications, unsupervised graph anomaly detection (UGAD) aims to identify abnormal data that significantly deviate from the majority of normal nodes without label annotations. Hence, UGAD can provide crucial assistance in enhancing the reliability of IoT, intelligent sensors and so on. Under the class-imbalanced reality caused by anomaly scarcity, the common paradigm of UGAD focuses on learning a model that primarily captures normal patterns. However, the traditional Graph Neural Network (GNN) paradigm suffers from local-aggregation limitations and over-smoothing, constraining their discrimination capacity. To address these issues, we introduce Graph Transformers (GTs) into UGAD task, termed as unsupervised attributed graph **A**nomaly detectio**N** w**I**th **M**asked gr**A**ph **T**ransform**E**rs (ANIMATE). Leveraging the global receptive field of Transformers, we can capture graph information that preserves the distinguishable characteristics of abnormalities from a global perspective. Furthermore, we employ masked auto-encoders to reconstruct node features and prompt our model to focus more on learning normal patterns. Additionally, we enhance the performance through a self-paced enhancement scheme specifically for UGAD tasks. Experiments conducted on various real-world benchmark datasets with organic anomalies validate the effectiveness of our proposed method compared to state-of-the-art competitors.

## 1. Introduction

Attributed graphs have emerged as a novel medium for representing information in numerous real-world sensors, incorporating nodes with entity features and edges denoting relationships between entities [1,2,3,4,5]. Graph-structured data is increasingly pivotal in data mining and signal processing, with graph anomaly detection (GAD) emerging as a compelling study area. GAD has been widely applied in real-world reliable sensors [6,7,8], including industrial process monitoring, cyber-physical intrusion detection, social spam detection, financial fraud detection, and so on. Given that labeling data is labor-intensive and time-consuming, exploring unsupervised graph anomaly detection (UGAD) solutions has become imperative. Therefore, this paper primarily focuses on unsupervised anomaly detection for discerning abnormal nodes within attributed graphs. Challenges have arisen due to the diversity of anomalies and the need for labeled data. To address these challenges, researchers endeavor to construct effective models that delineate normal patterns, enabling the detection of nodes that significantly deviate from these norms as anomalies.

Conventional GAD approaches traditionally utilize feature engineering or statistical models to differentiate between normal and abnormal entities, primarily focusing on modeling linear relationships. In recent years, Graph Neural Networks (GNNs) have emerged as a powerful paradigm for modeling the interactions between topology and attributes, becoming a dominant architecture in GAD tasks. Notably, DOMINANT [9] represents a pioneering framework that leverages Graph Convolutional Networks (GCNs) as the autoencoder backbone. This framework employs a shared GCN encoder alongside dual decoders to independently reconstruct node attributes and graph structures. Following this paradigm, subsequent studies [10,11] have widely adopted GCN-based autoencoders to characterize normal and abnormal patterns within graphs. Furthermore, models such as CONAD [12] employ graph augmentation and contrastive learning techniques built upon Graph Attention Networks (GATs). Similarly, GAD-NR introduces architectural flexibility by supporting diverse backbones, including GCN, GraphSAGE, and GIN. Ultimately, the overall efficacy of these methods fundamentally depends on the quality of the node representations extracted by the underlying GNN architectures.

**Figure 1:**
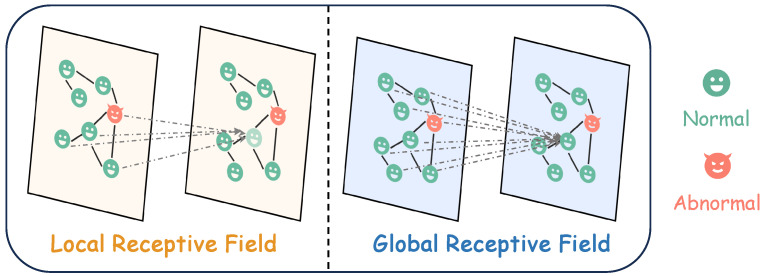
The comparison of the receptive field between GNNs (**left**) and Graph Transformers (**right**). The local aggregation of GNN-based networks may narrow the discrimination of normality/abnormality. With the natural imbalance of these components, the global receptive information brought by Graph Transformers contributes to capturing normal patterns for unsupervised graph anomaly detection.

Although GNNs have demonstrated satisfactory performance in detecting anomalies within graphs, they are not inherently well-suited for UGAD tasks due to their two prominent weaknesses [13], as shown in Figure 1. Firstly, the aggregation function employed in GNNs can obfuscate the model’s ability to discern normality from abnormality within attributed graphs. When an abnormal node is connected to normal nodes, the aggregation mechanism tends to smooth out anomalies within their local neighborhoods. Secondly, GNNs are prone to the over-smoothing problem, which restricts their capacity to go deeper. The over-smoothing problem limits their capability to move beyond local contexts and gather global information, which is essential for effective UGAD.

In fact, the presence of class-imbalanced data necessitates modeling normal patterns against which abnormal nodes can be identified. By utilizing global information, the model can prioritize most normal data instances and acquire high-quality representations. On the other hand, abnormal nodes, such as fake reviews, are usually scattered and lack normal neighbors, making them less suitable for local aggregation techniques. Therefore, incorporating global information can alleviate erroneous local aggregations on anomalies and their surrounding normal samples. Effectively capturing global information has emerged as a significant challenge in the field of UGAD that requires urgent attention and resolution.

Lately, Transformers have demonstrated remarkable ability in representation learning, which has generated a lot of interest and development in various fields, such as Natural Language Processing (NLP), Computer Vision (CV), and Time-series Analysis. The self-attention mechanism of Transformers allows them to perceive all other positions in the token. As a result, researchers are naturally inclined to incorporate Transformers into graph-structured data to enhance graph representation learning [14].

Inspired by graph Transformers and graph MAE [15], we propose a novel unsupervised attributed graph anomaly detection with masked graph Transformers, abbreviated as ANIMATE. Our version of ANIMATE is an encoder–decoder architecture based on the Transformers backbone. To alleviate the local aggregation problem, we employ Graph Transformers. As shown in Figure 1, compared to GNNs’ local receptive fields, Graph Transformers build a global receptive field via a self-attention mechanism. Global self-attention computes weights based on feature similarity rather than fixed edges. This allows anomaly nodes to assign low attention to dissimilar local neighbors and aggregate from similar distant nodes, effectively preventing feature smoothing between normal and abnormal. Additionally, the design of position embedding delays the aggregation of node features after judging the importance of neighbors. Consequently, Graph Transformers allow us to study the high-quality representation of normality and are naturally beneficial for UGAD tasks.

In addition, motivated by mask autoencoder methods, we utilize a masking strategy to enhance the difficulty of reconstructing abnormality. In the autoencoder fashion, the fundamental philosophy of the autoencoder is operating like a bottleneck to compress the data. The imbalanced nature of normality/abnormality tends to bias the autoencoder towards the majority class (i.e., normality) while eliminating sparse anomalies [16]. Monitoring the reconstruction error can differentiate normality and anomalies. In addition, anomalous nodes are a minority and deviate from their surroundings. When an anomalous node is masked, the surroundings are more likely to be normal nodes. Thus, the masking mechanism enhances the difficulty of reconstructing anomalies and enlarges the reconstruction loss gap between normality and anomalies. Meanwhile, with the reduction of node number, the memory consumption dilemma of Transformers can be relieved.

Overall, we aim to address the limitations of GNNs by combining Transformers and MAE. Specifically, we first randomly mask part of the nodes in the graph and then feed the visible graph into the encoder. The decoder tries to recover the masked nodes after gathering the low-dimensional embeddings. Additionally, we adopt Graphormer [14] as the crucial technique behind the encoder and decoder, which encourages the model to catch global topology information through well-designed positional encoding. To further construct a more precise model to describe normal patterns, we introduce a self-paced enhancement strategy to force the model to concentrate on the reconstruction of normality.

Most existing methodologies [9,10,12,17,18,19,20,21,22] only conduct their experiments on synthetic datasets, whose anomalies are injected artificially. However, Ref. [23] reveals that this widely used anomaly injection approach will cause data leakage issues. Therefore, we validate ANIMATE on real-world benchmark datasets with organic anomalies. In summary, the main contributions of this paper can be considered in terms of three aspects:

- For the first time, we introduce Graph Transformers for UGAD. The global information acquired by Transformers allows us to focus more on reconstructing normal patterns on the attributed graph.
- We determine that the masking node strategy is beneficial for UGAD tasks, which widen the differentiation of normal and abnormal nodes on reconstruction error. Moreover, we design a self-paced enhancement module to further refine the normality model that boosts performance.
- Comprehensive experiments on four real-world benchmark datasets with organic anomalies demonstrate the effectiveness of our proposed method compared to state-of-the-art baselines.

## 2. Related Work

### 2.1. Unsupervised Graph Anomaly Detection

With great potential in multi-media/multi-modal applications, UGAD receives considerable interest from both academia and industry. In the realm of multi-modal recommendations, leveraging graph anomaly detection can help distinguish anomalous nodes or edges that deviate from the user’s preferences or recommendation objectives. These anomalies might be data noise, fraudulent information, or illegal user behavior, necessitating timely identification and resolution. Hence, UGAD is a valuable topic in multi-media/multi-modal applications.

Reconstruction methods have always played an important role in the field of anomaly detection [16]. However, previous anomaly detection models cannot cope with the challenges brought by the complex structural information in graphs [24]. Ding et al. introduced a GCN-based [25] autoencoder into graph anomaly detection, termed DOMINANT [9]. It consists of a shared encoder and two types of decoders which handle the attribute information and the structural information separately. Subsequent endeavors have made more effective improvements based on GNN architecture. Ref. [10] changes the GCN to GAT [26], which can more accurately calculate reconstruction errors using the attention mechanism. ResGCN [27] makes an attempt to update the node representations with the residual information. Luo et al. [11] leverage the community information of nodes to improve performance. Ref. [23] proposes a new variance-based graph anomaly model, which can alleviate the issue of balanced detection. Furthermore, GAD-NR proposes a new neighborhood-reconstructed mechanism, which reconstructs the local structure, self-attributes, and neighbor attributes of a node [28].

### 2.2. Graph Transformer

A Transformer is a pioneering neural network architecture that has significantly improved the performance of various tasks in NLP [29]. The core idea of Transformers is the tailored multi-head self-attention mechanism, which allows the models to capture global information between subspaces. With a more comprehensive understanding of the context, the models can learn more powerful representations of data features. Inspired by the great success of Transformers in NLP, researchers have naturally employed them in other fields, e.g., CV and graph machine learning. So, a great deal of papers about Graph Transformers have emerged in recent years.

Aiming to address the drawbacks of over-reliance on connectivity relations and large-scale graphs limited by memory in GNNs, Zhang et al. [30] proposed a novel framework termed Graph-BERT, which does not require any graph convolution and aggregation operations. Different from the positional encoding style in Graph-BERT, Ref. [31] utilizes Laplacian eigenvectors to represent positional encoding, further improving the performance of the model in downstream tasks. Ref. [32] proposed a novel model named GraphiT, which combines graph structure and node positional information with the transformer architecture to achieve stronger representation capabilities than classical GNNs. In response to the difficulty in defining node positions in graph data, Ref. [33] designs a spectral attention network for learning node positions. Node features and positions are then fed into Transformers to learn node representations. The above models have achieved significant advancement in graph representation learning tasks.

In real-world applications, they are often limited by deep Transformer designs and the high memory consumption in large-scale graphs. Regarding this, Wu et al. [34] developed a framework named NodeFormer, which facilitates message passing between any two nodes and can be extended to large-scale graph node classification tasks. Furthermore, GMAE [15] takes advantage of masked autoencoder technology and Graph Transformer technology to address the aforementioned drawbacks. Through its delicate design, GMAE can serve as an effective pre-training facility in order to simplify the hard training process of Transformers. The mask-and-predict strategy reduces the number of input nodes, which relieves the memory consumption problem of Transformers.

However, for unsupervised graph anomaly detection, there is no sufficient attention on how to apply Graph Transformer models, which necessitates further research.

## 3. Notations and Problem Definitions

### 3.1. Notations

In this paper, $G=\left(V,E,X\right)$ is denoted as the attributed graph, $V=\{v_{1},v_{2}\ldots ,v_{N}\}$ indicates the node set of *N* nodes and $E=\{e_{ij}\}$ defines *M* edges. $e_{ij}=\left\{v_{i},v_{j}\right\}\in E$ indicates that there exists an edge connecting nodes $v_{i}$ and $v_{j}$. The adjacency matrix $A\in {\left\{0,1\right\}}^{N\times N}$ depicts the topological structure of G, where $A_{ij}=1$ if $e_{ij}\in E$; otherwise, $A_{ij}=0$. In addition, the node feature representations are represented as $X=\left[x_{1},x_{2}\ldots ,x_{N}\right]\in R^{N\times D}$, where $x_{i}$ denotes the *D*-dimension feature of node $v_{i}$.

### 3.2. UGAD Problem Formulation

In this paper, we focus on addressing the challenge of unsupervised node-level graph anomaly detection. This involves identifying nodes within an attributed graph that exhibit significant deviations from the majority pattern without any labeled information. The primary goal is to develop a scoring function F(·) that can assign an anomaly score $s_{i}$ to each node $v_{i}$ in the graph. A higher anomaly score suggests a higher likelihood of the node being an anomaly, while a lower anomaly score indicates a normal sample.

## 4. Method

**Figure 2:**
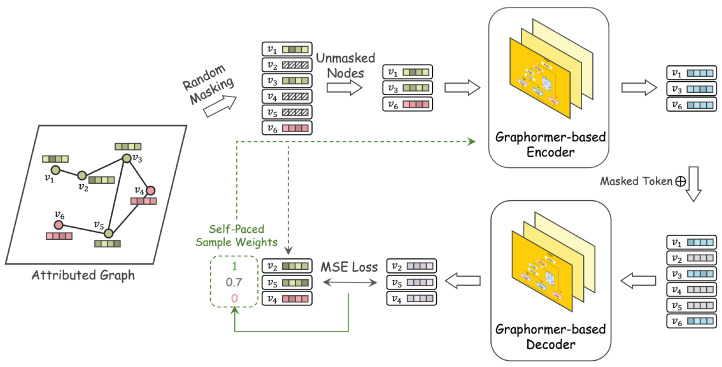
The training pipeline of our proposed ANIMATE. First, we randomly mask half of the nodes in the sample level. Next, we feed unmasked (visible) nodes into Graph Transformers with specially designed positional embedding, which aims to reconstruct the masked (invisible) node representation. Based on the ranking reconstruction loss, we calculate adaptive sample weights in a self-paced manner. This self-paced scheme gradually removes possible abnormal nodes in the masked counterparts and encourages the model to learn refinement normality.

In this section, we introduce the proposed ANIMATE algorithm in detail. As illustrated in Figure 2, ANIMATE operates in two stages, training and inference, under an unsupervised manner. Specifically, the training stage encompasses several steps: node masking, reconstruction based on graph transformers, and self-paced enhancement.

### 4.1. Graph Transformer with Position Embedding

#### 4.1.1. Basic Transformer

The Transformer architecture consists of a collection of several Transformer layers [29]. Among them, the self-attention module and position-wise feed-forward network (FFN) are the most important components in each Transformer layer. Given the input of self-attention module $H\in R^{n\times d}$, the Queries (Q), Keys (K), and Values (V) can be obtained from the following equation:

(1) $$Q=HW_{Q},\;K=HW_{K},\;V=HW_{V},$$

where *n* is the number of nodes, *d* is the input hidden dimension, and $W_{Q}\in R^{d\times d_{q}},W_{K}\in R^{d\times d_{k}},W_{V}\in R^{d\times d_{v}}$ are learnable matrices and usually $d_{q}=d_{k}$.

By calculating the similarity of queries and keys, we can acquire the weight of values and finally study the output representations of the self-attention module through

(2) $$\mathrm{Attention}\left(Q,K,V\right)=\mathrm{softmax}\left(\frac{QK^{T}}{\sqrt{d_{k}}}\right)V.$$

Next, we feed the node representations into a two-layer FFN in order to strengthen the learning capacity:

(3) $$\begin{array}{cc} \; & F_{1}\left(x_{i}\right)=\max\left(x_{i}W_{1}+b_{1},0\right),\\ \; & \mathrm{FFN}\left(x_{i}\right)=F_{1}\left(x_{i}\right)W_{2}+b_{2}, \end{array}$$

where $W_{1}$ and $W_{2}$ are learnable weights, and $b_{1}$ and $b_{2}$ denote bias.

#### 4.1.2. Positional Embedding for UGAD Task

**Figure 3:**
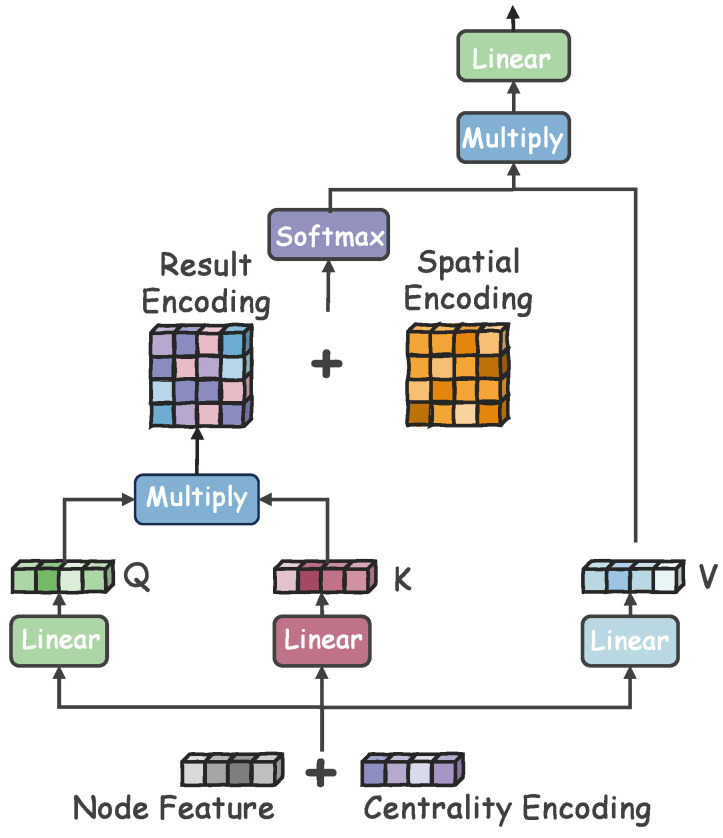
The architecture of the Graphormer-based encoder and decoder. Cooperating with centrality encoding and spatial encoding, the model can jointly capture the global topology information and contextual attributed information in graphs.

As the basic Transformer is designed for the NLP field, it is crucial for us to incorporate the structural characteristics of graphs into the Transformer architecture. Motivated by Graphormer [14], both the encoder and decoder components in ANIMATE are based on Graph Transformers with specially designed positional embedding. We exhibit the architecture of our encoder and decoder in Figure 3.

Concretely, we adopt centrality encoding and spatial encoding techniques in the Graphormer architecture as positional embeddings. To better understand the importance of nodes in the graph, we utilize centrality encoding. It emphasizes the semantic relationship between nodes regarding the node degrees.

In the GAD task, the degree centrality allows us to discover abnormalities in some cases. For example, in spam e-mail detection, the spam (i.e., abnormalities) usually has close connection edges with many users, which are shown to a high degree. As an additional signal to Transformers, centrality encoding is applied to each node by adding its in-degree and out-degree.

(4) $$h_{i}^{\left(0\right)}=x_{i}+z_{deg}^{-}\left(v_{i}\right)+z_{deg}^{+}\left(v_{i}\right),$$

where $z^{-},z^{+}\in R^{d}$ represents learnable embeddings of $v_{i}$ in-degree and out-degree, respectively. For undirected graphs, the in-degree and out-degree of nodes are same. In this way, both semantic correlation and node importance can be captured by the attention mechanism.

In Transformers, the token needs to explicitly specify its absolute or relative position. However, this is hard for graph data because the nodes in graphs are not organized in order as a sequence. Therefore, we cannot provide the ‘first’ node like sentences as positional information. To circumvent this crucial issue, we utilize spatial encoding to learn structural information in graphs. To be specific, we calculate the shortest path distances (SPDs) between all connected pairs of nodes. The function $\psi (v_{i},v_{j}):V\times V\to R$ denotes the spatial relationship between nodes $v_{i}$ and $v_{j}$ in graph G. If nodes $v_{i}$ and $v_{j}$ are connected, then the value equals the SPD; if not, we set it to be a special value, i.e., −1. Next, we index $\psi (v_{i},v_{j})$ into a learnable scalar, which serves as a self-attention bias $b_{\psi }$. The Equation (2) can be rewritten as

(5) $$\mathrm{Attention}\left(Q,K,V\right)=\mathrm{softmax}\left(\frac{QK^{T}}{\sqrt{d_{k}}}+b_{\psi }\right)V.$$

### 4.2. Masked Graph Transformer Reconstruction

Recently, the success of masked autoencoder-based methods in NLP paves a path for researchers of graphs [35]. Inspired by this simple yet effective approach, GraphMAE [36] introduces masked autoencoder techniques into graph pre-training models. It fixedly re-masks parts of nodes and builds a GIN-based encoder–decoder. Then, GraphMAE2 [37] enhances the model using a randomly re-masking strategy. Differently, S2GAE [38] employs a direction-aware masking strategy and tailors a cross-correlation decoder. HGMAE [39] extends the masked autoencoder technique to heterogeneous graph representation learning. However, there is still a relative dearth of research on the utilization of a masked autoencoder for GAD.

In our task, we consider that the MAE method suits graph anomaly detection for the following considerations. The majority of nodes are normal which causes the number imbalance phenomenon between normal and abnormal candidates. Most of the masking nodes are normal nodes, which helps the model focus more on reconstructing the patterns surrounding the normal nodes. Therefore, the masking mechanism helps to train a model that focuses on normal patterns, in which the reconstruction losses of normal nodes are small and vice versa.

Therefore, in the first step of ANIMATE, nodes within the input graph will be randomly masked with a certain ratio. Different from traditional GAD contrastive learning methods, which maximize the respective correlation [19,21,40,41], we adopt the reconstruction paradigm without the additional expense of augmented view calculation. Subsequently, the unmasked nodes are passed through the encoder to derive their embeddings. These masked nodes are excluded from the encoder’s view, which solely processes the features of the visible nodes to generate embeddings for each of them. Then, a common learnable mask token is employed to signify the embeddings of the masked nodes, which are then integrated into the encoder’s output. Finally, the resulting embedding matrix, inclusive of the inserted mask tokens, is fed into the decoder to reconstruct the characteristics of the masked nodes.

Following the instruction of [9], the abnormality can be reflected by the reconstruction loss. Compared to directly reconstructing the node attribute, we introduce a more challenging task that utilizes the unmasked node information to reconstruct masked nodes. The reconstruction loss of masked node $v_{i}$ can be calculated by

(6) $$L_{re}{\left(v_{i}\right)}_{masked}={∥\phi \left(x_{i}\right)-x_{i}∥}^{2},$$

where ϕ(·) is the graph transformer network.

### 4.3. Self-Paced Enhancement

As mentioned before, the essential goal of the UGAD mechanism is to learn a normality model [41]. The node that deviates significantly from this model can be regarded as an abnormality. Since there is no label information during training, the irregular anomalous nodes hinder ANIMATE from learning the ideal normality model. To alleviate this problem, a natural solution is to force our model to pay more attention to the reliable normal pattern, and mitigate the impact of the suspicious anomalous pattern. To this end, we aim to refine the normality model via self-paced learning (SPL) [42].

Traditional SPL intends to incrementally increase hard samples in an easy-to-complex manner. However, we aim to gradually remove dubious abnormal nodes in the training phase according to the ranked reconstruction loss. Therefore, we design a self-paced enhancement (SPE) strategy for UGAD tasks to further improve the performance. The nodes with smaller ranked reconstruction loss are more likely to be normal nodes. Consequently, instead of learning a UGAD model adopting all samples, we adaptively assign higher weights to more credible normal nodes in a self-paced manner. To be specific, in each iteration, SPE selects suspicious anomalous nodes with large reconstruction loss based on the already learned model and allocates smaller weights for them to be used in the subsequent iterations.

Concretely, SPE assigns learnable weight $\omega =\left[\omega_{1},\omega_{2}\ldots ,\omega_{N}\right]\in {\left[0,1\right]}^{N}$ for each node in the dataset, and the optimal goal of SPE can be realized by the following function:

(7) $$L_{SPE}=\sum_{i=1}^{N}\omega_{i}L\left(v_{i}|\theta \right)+f\left(\omega |\xi \right),$$

where $L(v_{i}|\theta )$ represents the reconstruction loss $L_{re}{\left(v_{i}\right)}_{masked}$ in Equation (6) and θ represents the parameters of our graph transformer model, f(ω|ξ) is a self-paced regularizer, and ξ is the age parameter that controls the learning pace. Through the alternative search scheme (ASS) [42], we can solve Equation (7) by alternatively optimizing either θ or ω while fixing the other. When ω is fixed, the optimization goal is transformed into

(8) $$\min_{\theta }\sum_{i=1}^{N}\omega_{i}L\left(v_{i}|\theta \right).$$

Equation (8) can be optimized via gradient descent. When θ is fixed, the objective function can be written as

(9) $$\min_{\omega }\sum_{i=1}^{N}\omega_{i}L\left(v_{i}|\theta \right)+f\left(\omega |\xi \right).$$

We expect to embed mixture weights for each node in Equation (9). Should the reconstruction loss of a node be excessively high or low, the node is likely to be an anomaly or normal, respectively. Hence, we directly set the self-paced weight $\omega_{i}$ to be 0 or 1. For other conditions, the self-paced weights and reconstruction losses should be negatively correlated. Inspired by [43], we adopt a mixture self-paced regularizer strategy to search for the optimal sample weight:

(10) $$f\left(\omega |\xi ,\xi^{\prime }\right)=-\delta \sum_{i=1}^{N}\ln\left(\omega_{i}+\frac{\delta }{\xi }\right),$$

where $0<\xi^{\prime }<\xi$ and $\delta =\xi \xi^{\prime }/\left(\xi -\xi^{\prime }\right)$ are additional parameters. Since the above regularizer is convex, the partial gradient of Equation (9) equals

(11) $$\frac{\partial L_{SPE}}{\partial \omega_{i}}=L\left(v_{i}|\theta \right)-\frac{\xi \delta }{\xi \omega_{i}+\delta }=0,\;i=1,2,\ldots ,N.$$

The closed-form optimal solution for the self-paced weights can be calculated as follows:

(12) $$\omega_{i}^{*}=\left\{\begin{array}{cc} 1, & L\left(v_{i}|\theta \right)\leq \xi^{\prime },\\ \frac{\delta }{L(v_{i}|\theta )}-\frac{\xi^{\prime }}{\xi -\xi^{\prime }}, & \xi^{\prime }<L\left(v_{i}|\theta \right)<\xi ,\\ 0, & L(v_{i}|\theta )\geq \xi . \end{array}\right.$$

In this way, the self-paced weights are highly associated with reconstruction loss, i.e., the anomalous degree of nodes, which is specifically designed for the UGAD task. To determine the value of ξ and $\xi^{\prime }$ in an adaptive manner, we let them change according to the reconstruction loss varying across iterations. The lower threshold $\xi^{\prime }$ controls the number of ‘normal nodes’ that contribute to the learning progress of the normality model. Due to normal nodes constituting a relatively major proportion of the dataset, we expect the number of $\xi^{\prime }$ to be large. We first calculate the mean μ(t) and standard deviation σ(t) of node reconstruction loss at *t*-th iteration of model training. Next, we set $\xi^{\prime }=\mu \left(t\right)+\sigma \left(t\right)$. The upper threshold ξ helps the model to relieve the negative effects caused by suspicious abnormal samples. So we set it as follows:

(13) $$\xi =max\{\mu \left(t\right)+\left(4-t\cdot s\right)\cdot \sigma \left(t\right),\xi^{\prime }\},$$

where *s* is the shrink rate.

After labeling, we only calculate the reconstruction loss of nodes that are labeled as normal. As for unsupervised graph anomaly detection tasks, the abnormalities are rare and various, rendering the generation of a typical classification model impractical. Normally, the aim of our method is to capture normal patterns, and therefore, we only reconstruct the normal nodes while ignoring others.

### 4.4. Inference Stage

It is worth noting that ANIMATE is an unsupervised transductive solution that directly detects anomalies from the testing set. Therefore, after training, we infer the anomaly score of each node by the respective reconstruction loss.

## 5. Experiments and Discussion

### 5.1. Datasets

We conduct extensive experiments using four benchmark datasets to verify the validity of ANIMATE. It is worth noting that we perform the experiments on real-world datasets containing real anomalies but not synthetic datasets with injected anomalies. As pointed out in [23], the current widely used anomaly injection approach suffers from a serious data leakage issue. Therefore, we only carry out our experiments on real datasets with organic anomalies. Detailed statistical information is shown in Table 1.

- **Disney** [44]: Disney is a co-purchase network of movies from the Amazon network, in which the anomaly label is obtained by voting from high school students.
- **Books** [45]: Books is a graph dataset about books purchased from the Amazon network, in which the anomaly label is determined based on the *amazonfail* tag information that is provided by Amazon users.
- **Reddit** [46]: Reddit is a social network dataset derived from the social media platform Reddit where users banned from Reddit sites are flagged as anomalous users.
- **Yelp** [47]: Yelp contains restaurant reviews from several states in the U.S. where there is a connecting relationship between reviews posted by the same user. The anomaly tag is a fake review.

### 5.2. Comparison Algorithms

We have chosen three classical graph anomaly detection algorithms and eight deep learning-based graph anomaly detection algorithms as our competitors.

- **LOF** [48]: Local Outlier Factor(LOF) measures the difference between a node and its neighborhood, where the larger the difference, the more abnormal the node is.
- **SCAN** [49]: SCAN utilizes graph structure information for clustering to find outliers.
- **Radar** [50]: Radar adopts residual decomposition to identify anomalous nodes with significant residual errors.
- **DOMINANT** [9]: DOMINANT consists of three parts, namely a shared GCN encoder, an attribute decoder, and a structure decoder, which work together to reconstruct the raw features. The larger the reconstruction error, the more anomalous the node.
- **DONE** [51]: DONE mainly contains structure and attribute autoencoders.
- **CoLA** [21]: CoLA introduces the node–subgraph contrastive strategy, which distinguishes the node that is not similar to its corresponding subgraph as an anomaly.
- **ANEMONE** [19]: ANEMONE is a multi-scale contrastive graph anomaly detection framework consisting of node–node contrastive and node–subgraph contrastive.
- **NLGAD** [41]: NLGAD proposes a graph anomaly detection framework based on normality learning to enhance representation learning of normal nodes.
- **VGOD** [23]: VGOD detects structure and attribute anomalies by calculating the variance between nodes and their neighborhoods and combining the node attribute with the reconstruction module.
- **PREM** [52]: PREM designs the attribute pre-processing module and ego-neighbor matching network and adopts contrastive learning mode to train.
- **GRADATE** [40]: GRADATE introduces edge perturbation for graph augmentation and designs a subgraph–subgraph contrastive mode.
- **GADAM** [53]: GADAM decouples local inconsistency mining from message passing and further detects anomalies beyond local scope via adaptive message passing and global consistency discernment.

### 5.3. Parameter Settings

To balance performance and complexity, we set the number of encoder layers to 6 and the number of decoder layers to 2. The features of all nodes are mapped to 256 dimensions. The masking ratio is set to 50%. The batch size during model training is 128. The shrink rate *s* is usually set to be small, and we adopt 0.0001 for each dataset. We train the model for Disney, Books, Reddit, and Yelp datasets with 500, 300, 100, and 50 epochs, respectively. And the learning rate of the model on Disney, Books, Reddit, and Yelp is $1\times 10^{-3}$, $1\times 10^{-3}$, $1\times 10^{-6}$, and $2\times 10^{-6}$, respectively.

### 5.4. Experimental Analysis

We adopt ROC-AUC (AUC) as the model performance evaluation metric. Table 2 shows the AUC values of our proposed model, ANIMATE, and eleven comparative algorithms.

Analyzing the results, we can draw the following conclusions:

- Our proposed model, ANIMATE, consistently achieves the best performance on the four real-world datasets. In particular, ANIMATE outperforms the second best comparison algorithm by a notable margin, i.e., **16.11**% and **9.89**% on the Books and Yelp datasets, respectively. These results also demonstrate that utilizing Transformers and MAE as backbone is a promising solution for UGAD tasks.
- The proposed ANIMATE algorithm is capable of extending to large-scale datasets. Specifically, for the Yelp dataset, most deep learning-based graph anomaly detection algorithms suffer from exceeding memory. This is due to the reason that ANIMATE is designed using a batch manner instead of inputting the whole graph. The extensibility of ANIMATE is flexible.
- Compared with contrastive-based graph anomaly detection methods, including CoLA, ANEMONE, SL-GAD, NLGAD, and GRADATE, our proposed generative-based solution enables us to obtain notable AUC growth. Owing to these methods, we focus on changes between nodes and their neighborhoods while ignoring node global long-range dependency information.

### 5.5. Parameter Sensitivity Analysis

In this subsection, we analyze the sensitivity of key parameters to further investigate our proposed model in terms of the masking ratio, the number of epochs, and the embedding dimension.

**Figure 4:**
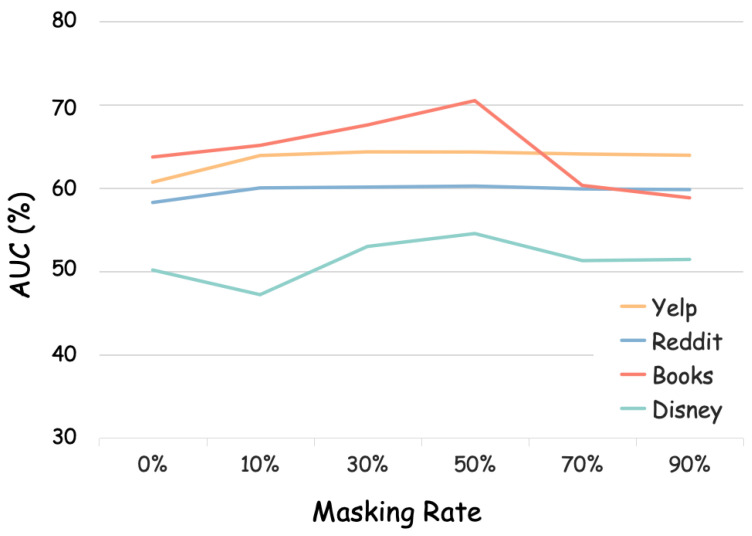
Parameter study of masking rate w.r.t. AUC on benchmark datasets.

First, we study how the **masking rate** affects the performance of our proposed method. In practice, we conduct the experiments under different masking rate settings, which vary from no masking (0%) to 90% nodes masked. Figure 4 indicates that ANIMATE exhibits the best detection capability when half of the nodes are randomly masked. In addition, the performance under the 50% masking rate setting consistently outperforms no masking on all datasets, which aligns with mainstream observations in related works [15,36,54]. This phenomenon demonstrates the effectiveness of the masking mechanism on the UGAD task. Moreover, masking ratio is related to information redundancy in graphs. When the masking ratio is too low, it means that only a small proportion of samples are reconstructed. And when the masking ratio is too high, it will be difficult to recover node information. Both of these circumstances are not beneficial for discovering abnormality and normality. So, it is important to select an appropriate masking rate in the MAE framework. In ANIMATE, we fix the value of the masking rate to be 50% on all datasets.

**Figure 5:**
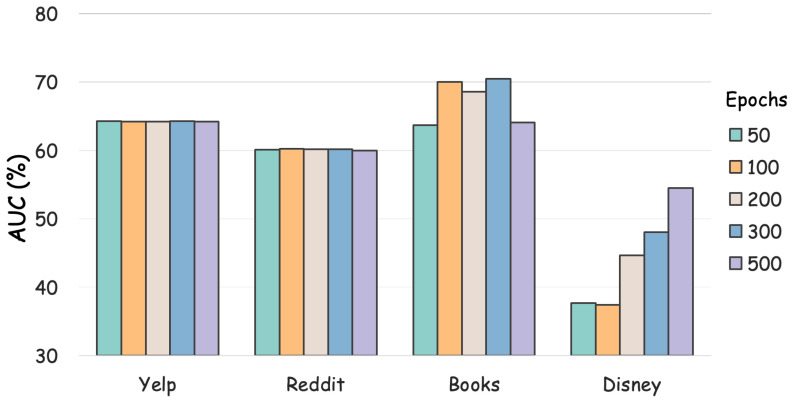
Effect of different epoch settings on AUC value.

We further investigated the impact of the number of **epochs** on detection performance. Specifically, we modify the value of training epochs as 50, 100, 200, 300 and 500. The performance variance results are visualized in Figure 5. It can seen that our proposed method is stable on the Yelp and Reddit datasets with different numbers of epochs. ANIMATE slightly fluctuates on Books and Disney. The possible underlying reason is that the scale of the Books and Disney datasets is relatively small.

Additionally, we also conduct experiments to assess the influence of the **embedding dimension** on our proposed model. The AUC scores over different choices of latent dimension are reported in Table 3. The embedding dimension is altered in the range of {32, 64, 128, 256, 512}. We observed that, for all four datasets, 256 dimensions of latent space provides valuable information without redundancy for the UGAD task. As a result, we set the latent parameter to be 256 to achieve balance between efficiency and effectiveness.

### 5.6. Ablation Study

To demonstrate the magnitude of the self-paced enhancement strategy for UGAD tasks, we perform ablation study experiments with and without SPE under unsupervised settings. As shown in Table 4, SPE brings 0.49–20.19% AUC gains on four datasets. In particular, the performance improvement on the Disney dataset is conspicuous. The results verify that SPE allows our method to pay more attention to normal samples and build a high-quality normality model.

## 6. Conclusions

In this paper, we propose a novel unsupervised attributed graph anomaly detection with masked Graph Transformers, termed ANIMATE. It is an encoder–decoder architecture based on the Graphormer backbone. To further learn a high-quality normal pattern, we introduce a self-paced strategy based on ranking reconstruction loss to force the model to concentrate on recovering normality. In the future, we will explore how to further improve the architecture of Transformers for UGAD tasks.

## Figures and Tables

**Table 1:** The statistics of the datasets.

Dataset	#Nodes	#Edges	#Attributes	#Anomalies	#Anomalous Ratio (%)
**Disney**	124	335	28	6	4.84
**Books**	1418	3695	21	28	1.97
**Reddit**	10,984	168,016	64	366	3.31
**Yelp**	45,954	3,846,979	32	6677	14.53

**Table 2:** AUC(%) performance on four real-world benchmarks with organic anomalies. The best result in each experiment is in **bold**. The suboptimal result is underlined. OOM_G denotes out of CUDA memory and OOM_R denotes out of RAM memory.

Category	Methods	Disney	Books	Reddit	Yelp
Classical	LOF (SIGMOD, 2000)	47.88 ± 0.0	36.52 ± 0.0	57.16 ± 0.0	54.41 ± 0.0
	SCAN (SIGKDD, 2007)	49.33 ± 4.0	49.19 ± 1.7	49.56 ± 0.3	47.98 ± 2.7
	Radar (IJCAI, 2017)	51.84 ± 0.0	52.18 ± 0.0	56.10 ± 1.2	OOM_G
Deep	DOMINANT (SDM, 2019)	51.12 ± 3.0	46.01 ± 2.7	55.35 ± 0.1	OOM_G
	DONE (WSDM, 2020)	44.17 ± 6.2	41.92 ± 4.0	54.59 ± 2.9	OOM_G
	CoLA (TNNLS, 2021)	35.03 ± 7.6	52.37 ± 4.2	55.52 ± 1.1	48.04 ± 3.9
	ANEMONE (CIKM, 2021)	44.35 ± 4.1	54.37 ± 3.7	53.45 ± 1.4	46.97 ± 4.4
	NLGAD (ACM MM,2023)	33.19 ± 4.8	50.95 ± 2.1	58.93 ± 0.8	46.77 ± 2.5
	VGOD (ICDE, 2023)	27.82 ± 1.3	36.71 ± 3.6	52.67 ± 0.7	OOM_G
	PREM (ICDM, 2023)	39.55 ± 3.6	43.07 ± 2.3	41.19 ± 1.6	OOM_G
	GRADATE (AAAI, 2023)	44.20 ± 2.7	54.12 ± 3.2	58.77 ± 2.1	OOM_R
	GADAM (ICLR, 2024)	39.66 ± 2.2	53.28 ± 2.8	47.20 ± 1.5	52.89 ± 3.1
	**ANIMATE (Ours)**	**53.81** ± 1.9	**70.48** ± 2.1	**60.22** ± 0.2	**64.30** ± 2.4

**Table 3:** Influence of different embedding dimensions of Graphformer on AUC(%) performance. The best performance is in **bold**.

Embedding Dimension	32	64	128	256	512
**Disney**	49.58	50.28	48.87	**53.81**	50.14
**Books**	64.77	55.97	66.20	**70.48**	66.37
**Reddit**	56.81	54.26	53.74	**60.22**	55.17
**Yelp**	56.27	55.62	54.05	**64.30**	59.03

**Table 4:** The ablation study of self-paced enhancement (SPE). Specifically, ✗denotes the performance of ANIMATE without the SPE module and ✓denotes its performance incorporating the SPE module.

SPE	Disney	Books	Reddit	Yelp
✗	33.62	67.87	59.73	60.86
✓	**53.81**	**70.48**	**60.22**	**64.30**

## Data Availability

Data will be made available on request.

## Abbreviations

The following abbreviations are used in this manuscript:

UGAD	Unsupervised graph anomaly detection
GNNs	Graph neural networks
AUC	Area under curve
OOM_G	Out of cuda memory
OOM_R	Out of RAM memory
